# Elevated C-reactive protein, interleukin 6, tumor necrosis factor alpha and glycemic load associated with type 2 diabetes mellitus in rural Thais: a cross-sectional study

**DOI:** 10.1186/s12902-017-0189-z

**Published:** 2017-07-17

**Authors:** Chanchira Phosat, Pornpimol Panprathip, Noppanath Chumpathat, Pattaneeya Prangthip, Narisara Chantratita, Ngamphol Soonthornworasiri, Somchai Puduang, Karunee Kwanbunjan

**Affiliations:** 1 Department of Tropical Nutrition and Food Science, Faculty of Tropical Medicine, Mahidol University, 420/6 Ratchawithi Rd, Bangkok, 10400 Thailand; 2 Faculty of Nursing, Huachiew Chalermprakiet University, 18/18 Bangna-Trad Rd, Samut Prakan, 10540 Thailand; 3 Department of Microbiology and Immunology, Faculty of Tropical Medicine, Mahidol University, 420/6 Ratchawithi Rd, Bangkok, 10400 Thailand; 4 Department of Tropical Hygiene, Faculty of Tropical Medicine, Mahidol University, 420/6 Ratchawithi Rd, Bangkok, 10400 Thailand

**Keywords:** Type 2 diabetes mellitus, CRP, IL6, TNF-α, Glycemic load

## Abstract

**Background:**

The elevated levels of inflammatory markers, including C-reactive protein (CRP), tumor necrosis factor alpha (TNF-α), and interleukin 6 (IL6) are supposed to be associated with type 2 diabetes mellitus (T2DM). Frequent high glycemic load (GL) consumption, central obesity, and a lack of physical activity are considered to be T2DM risk factors. This study aimed to determine the difference of these inflammatory markers as well as GL in individuals with versus those without T2DM in rural Thais.

**Methods:**

A total of 296 participants aged 35–66 living in Sung Noen District, Nakhon Ratchasima Province, Thailand, were recruited. Blood was collected to evaluate blood glucose levels, lipid profiles, and inflammatory markers. A Semi-food frequency questionnaire was utilized to assess GL followed by socioeconomic and anthropometric assessment. Statistical analysis was subsequently performed.

**Results:**

Elevated CRP and IL6 levels were associated with increased risk of developing T2DM [OR (95% CI): 7.51 (2.11, 26.74) and 4.95 (1.28, 19.11)], respectively. There was a trend towards increased risk of T2DM with elevated TNF-α levels [OR (95% CI): 1.56 (0.39, 6.14)]. GL correlated significantly with fasting blood glucose (*r* = 0.289, *P* = 0.016), suggesting that it is involved in T2DM in this study group.

**Conclusion:**

In this study, CRP, IL6, and TNF-α associated with T2DM. Our findings suggested that these inflammatory markers, especially CRP, may initiate T2DM.

**Electronic supplementary material:**

The online version of this article (doi:10.1186/s12902-017-0189-z) contains supplementary material, which is available to authorized users.

## Background

Diabetes is the second-most prevalent non-infectious disease in Thailand [1]. A high morbidity rate has been found in the North-Eastern region, especially in those aged over 35 [2].

Effects of the inflammatory markers such as C-reactive protein (CRP), tumor necrosis factor alpha (TNF-α) and interleukin 6 (IL6) that are triggered by excessive adipose tissue have been reported on insulin signaling pathways, resulting in insulin resistance and eventually progressing to type 2 diabetes mellitus (T2DM) [3, 4].

Elevation of CRP was related to an increased risk of developing T2DM [5, 6] and was suggested as an independent risk determinant for newly diagnosed T2DM [7, 8]. However, these results are controversial. Some studies stated a link between CRP and increased risk of prolonged T2DM development [9].

TNF-α was believed to induce insulin resistance by inhibiting phosphorylation of IRS-1 and Akt substrate 160 on insulin signaling cascade [10, 11]. A previous study also revealed the role of TNF-α in reducing insulin production from β-cell [12]. Therefore, this marker is suspected to be a possible mediator between insulin resistance and diabetes.

IL6 level was found to develop in accordance with glucose and insulin concentration [13]. It was reported to impact glucose homeostasis and metabolism and possibly acts indirectly on adipocytes and the pancreatic β-cell [14]. The relationship between IL6 and T2DM was revealed in several studies [13, 15, 16]. However, its role as a predictive marker for T2DM is still suspected.

In addition to inflammatory markers, inadequate diet is deemed to be a T2DM risk factor. There are reports regarding noteworthy associations of insulin resistance with high glycemic load (GL) diets [17–20]. GL was found to rapidly increase both blood sugar and insulin levels [17, 20]. Those with high GL revealed a greater trend of T2DM incidence than those with low GL [17–20].

Identifying predictive factors for T2DM will be beneficial to developing effective prevention and early detection of the disease. Therefore, the objectives of this study were to evaluate whether inflammatory markers and carbohydrate consumption, indicated by glycemic load (GL), were different in individuals with versus without T2DM.

## Methods

### Study subjects

Men and women aged 35–66 were recruited in Sung Noen District, Nakhon Ratchasima Province, Thailand, an area transitioning from a rural to an industrial society. Exclusion criteria were pregnancy or lactation, diagnosis of any chronic disease, regular medicine use, and the presence of infection. A total of 296 subjects, consisting of 100 men and 196 women, were recruited. The study procedure was approved by the Ethics Committee of the Faculty of Tropical Medicine, Mahidol University (TMEC 16–042). Written informed consent was obtained from all participants. Socioeconomic information and physical activity data were collected, and blood pressure was measured.

### Anthropometric assessment

Subjects underwent anthropometric assessment to gauge individual health status. Weight and height were measured and body mass index (BMI) was calculated. To assess abdominal obesity, waist circumference (WC) was measured at the midpoint between the lower rib and iliac crest. A body composition monitor (HBF-375, Omron Healthcare, Japan) was used to determine each subject’s body composition, including percentage of body fat (BF), visceral fat (VF), trunk fat (TrF), and muscle mass.

### Dietary assessment

Food consumption patterns were assessed using a validated semi-food frequency questionnaire (semi-FFQ) (Additional file 1). Usual food consumption in the study area was surveyed before creating food items, with 162 food items listed in total. The amount of food intake was divided into three groups: less than, equal to, or more than standard portion size based on the Thai food exchange list [21]. Nine frequency categories ranging from never or less than once/month to six times/day were used. Subjects’ individual daily energy intake was estimated using the NutriSurvey program (Copyright© 2007, SEAMEO-TROPMED RCCN-University of Indonesia). To estimate the effect of food intake on blood glucose levels, GL calculations were performed by multiplying the approximate amount of daily consumed carbohydrate in grams by the reference glycemic index [22] then dividing by 100.

### Physical activity assessment

Subjects reported their frequency of regular activity during the previous month. The obtained data was used to determine the metabolic equivalent of task (MET) value [23], and the average total daily energy expenditure (TDEE) was calculated using the following equation: TDEE (kcal) = MET (kcal*kg^−1^*h^−1^) × weight (kg) × time (h).

### Laboratory analysis

Blood samples were collected after fasting for 12 h to evaluate concentrations of biochemical parameters.

#### Assessment of blood glucose and insulin

Fasting blood glucose (FBG), glycohemoglobin (HbA_1c_), and fasting insulin were assessed using whole blood collected at baseline of an oral glucose tolerance test (OGTT). Two-hour blood glucose (2hBG) was assessed using whole blood collected two hours after the oral administration of 75 g of glucose. FBG, HbA_1c_, and 2hBG were measured by a Cobas® 6000 analyzer (Roche Diagnostics Ltd., Switzerland). Fasting insulin level was measured with a human insulin ELISA kit (EMD Millipore, Billerica, USA). Fasting glucose and fasting insulin levels were then used to estimate insulin resistant state and β-cell function as follows [24]:

Homeostatic model assessment of insulin resistance (HOMA-IR):

HOMA-IR = [Fasting insulin (μIU/mL) × Fasting glucose (mmol/L)] / 22.5.

Homeostatic model assessment of β-cell function (HOMA-β):

HOMA-β = [20 × Fasting insulin (μIU/mL)] / [Fasting glucose (mmol/L) – 3.5].

#### Assessment of lipid profiles

The enzymatic colorimetric method was performed using a Cobas® 6000 analyzer (Roche Diagnostics Ltd., Switzerland) to determine triglyceride (TG), total cholesterol (TC), and high density lipoprotein cholesterol (HDL-c) levels. Low density lipoprotein cholesterol (LDL-c) level was determined using the Friedewald equation [25] as follows: LDL-c = total cholesterol – (HDL-c + TG / 5).

#### Determination of concentration of inflammatory markers

A nephelometer (Siemens Healthcare GmbH, Germany) was used to analyze CRP levels while TNF-α and IL6 were evaluated using an ELISA kit (Human IL6/TNF ELISA set, BD Biosciences, California).

### Statistical analysis

Before conducting statistical analysis, study subjects were divided into three groups according to their blood glucose levels based on the 2006 diagnostic criteria of the World Health Organization, as follows. Normal group: FBG ≤ 100 mg/dl, OGTT ≤ 139 mg/dl, and HbA_1c_ ≤ 5%; pre-diabetic group: FBG 100–125 mg/dl, OGTT 140–199 mg/dl, or HbA_1c_ 5.1–6.4%, and T2DM group: FBG ≥ 126 mg/dl, OGTT ≥ 200 mg/dl, or HbA_1c_ ≥ 6.5%. All statistical analysis was performed by SPSS version 18. Differences between study groups were determined by Kruskal-Wallis and Mann-Whitney U tests. Socioeconomic data, frequency of physical activity, smoking status, and alcohol consumption status were compared using chi-square tests. Correlation between variables was tested by Spearman’s Rank correlation. The association between inflammatory markers and T2DM was estimated by odds ratios and 95% confidence intervals (CI), which were obtained from logistic regression. A *P* value <0.05 was considered statistically significant.

## Results

The normal, pre-diabetic, and T2DM groups consisted of 51 subjects (14 male, 37 female), 204 subjects (74 male, 130 female), and 41 subjects (12 male, 29 female) respectively. A comparison of socioeconomic data and other risk factors for T2DM for normal vs. pre-diabetic and T2DM groups is shown in Table 1. Approximately two thirds of each study group did not have a family history of diabetes. Most subjects finished primary school. Only 1.5% of those in the pre-diabetic group completed a bachelor degree. The most common occupation in all groups was freelancer/factory worker followed by farmer. Due to their occupation, around 50% of each group was physically active 5 times a week or more. Most subjects did not smoke and almost half did not drink. Overall, educational level was low to moderate.

**Table 1:** Socioeconomic characteristics and risk factors for type 2 diabetes mellitus (T2DM) according to study group

	Normal group n (%)	Pre-diabetic group		T2DM group	
		n (%)	*P* ^a^	n (%)	*P* ^b^
Family history of diabetes			0.352		0.468
No	32 (68.1%)	148 (74.7%)		23 (60.5%)	
Yes	15 (31.9%)	50 (25.3%)		15 (39.5%)	
Educational level			0.463		0.198
Uneducated	3 (6.1%)	11 (5.5%)		0 (0.0%)	
Primary school	36 (73.5%)	159 (79.1%)		34 (85.0%)	
High school	8 (16.3%)	26 (12.9%)		3 (7.5%)	
Vocational college	2 (4.1%)	2 (1.0%)		3 (4.5%)	
Bachelor degree	0 (0.0%)	3 (1.5%)		0 (0.0%)	
Occupation			0.592		0.806
Farmer	17 (33.3%)	82 (40.4%)		17 (42.5%)	
Freelancer or factory worker	28 (54.9%)	86 (42.4%)		19 (47.5%)	
Grocer	3 (5.9%)	16 (7.9%)		2 (5.0%)	
Housekeeper	2 (3.9%)	11 (5.4%)		2 (5.0%)	
Others	1 (2.0%)	8 (3.9%)		0 (0.0%)	
Frequency of physical activity			0.280		0.362
Never	17 (33.3%)	53 (26.1%)		9 (22.5%)	
1–2 times/week	3 (5.9%)	25 (12.3%)		6 (15.0%)	
3–4 times/week	7 (13.7%)	17 (8.4%)		4 (10.0%)	
5 times/week or more	24 (47.1%)	108 (53.2%)		21 (52.5%)	
Smoking status			0.280		0.193
Never smoke	36 (70.6%)	147 (72.0%)		31 (77.5%)	
Still smoke	14 (27.5%)	42 (20.7%)		6 (15.0%)	
Used to smoke	1 (2.0%)	14 (6.9%)		3 (7.5%)	
Alcoholic drinking status			0.261		0.286
Never drink	24 (48.0%)	98 (48.3%)		22 (55.0%)	
Still drink	25 (50.0%)	88 (43.3%)		15 (37.5%)	
Used to drink	1 (2.0%)	17 (8.4%)		3 (7.5%)	

*P* values were calculated by chi-square test for the differences between groups

^a^Normal group vs. pre-diabetic group

^b^Normal group vs. type 2 diabetic group

Anthropometric and clinical characteristics are presented in Table 2. The age ranges of the study groups showed no statistical difference. All anthropometric parameters including BMI, WC, BF, VF, and TrF were significantly different between groups except muscle mass percentage. These parameters represent the risk of developing T2DM. The T2DM and pre-diabetic groups had statistically higher levels of both systolic and diastolic blood pressure than the normal group. Significant differences were also found in insulin resistance parameters including insulin level, HOMA-IR, and HOMA-β. Additionally, among lipid profiles, a marked difference in TG was observed between study groups.

**Table 2:** Anthropometric and clinical characteristics of subjects according to study group

	Normal group (*n* = 51)	Pre-diabetic group (*n* = 204)	T2DM group (*n* = 41)	*p*
Age (years)	44 (36,55)	45 (34,60)	46 (35,66)	0.131
BMI (kg/m^2^)	23.30 (17.30,37.60)	25.70 (16.60,43.00)^a^	27.20 (19.00,43.00)^b,c^	0.000
WC (cm)	78.50 (63.50,110.50)	84.00 (61.50,130.00)	90.50 (74.00,108.00)^b,c^	0.000
Body fat (%)	30.00 (14.90,41.70)	31.15 (9.50,72.00)	34.15 (19.30,42.80)^b,c^	0.018
Visceral fat (%)	7.00 (1.50,22.00)	8.50 (1.00,83.00)^a^	11.00 (4.50,27.00)^b,c^	0.000
Trunk fat (%)	21.70 (8.50,37.20)	23.15 (5.50, 78.00)	26.60 (11.00,39.00)^b,c^	0.021
Muscle (%)	25.00 (21.90,33.60)	25.10 (19.60,84.00)	23.90 (20.40,31.70)	0.060
Systolic BP (mmHg)	111 (75,139)	120 (71,159)^a^	127 (95,154)^b,c^	0.000
Diastolic BP (mmHg)	72 (49,111)	76.50 (23, 109)^a^	79 (68,102)^b,c^	0.000
Insulin (μU/mL)	4.64 (0.04,8.46)	5.83 (0.46,32.94)^a^	6.29 (0.75,46.66)^b^	0.002
HOMA-IR	1.03 (0.01,1.94)	1.24 (0.11,7.48)^a^	1.80 (0.15,18.73)^b,c^	0.000
HOMA-β	66.06 (0.66,163.39)	74.18 (5.59,410.21)	52.62 (5.23,186.95)^b,c^	0.003
TG (mg/dl)	93.00 (52.00,273.00)	128.00 (54.00,735.00)^a^	146.00 (64.00,460.00)^b^	0.000
TC (mg/dl)	192.00 (95.00,306.00)	200.00 (100.00,388.00)	206.00 (92.00,290.00)	0.438
HDL-c (mg/dl)	54.00 (20.00, 83.00)	51.00 (10.00,91.00)	46.00 (4.00,143.00)	0.181
LDL-c (mg/dl)	119.00 (37.00,223.00)	120.00 (8.00,322.00)	122.00 (35.00,207.00)	0.966

Data presented as median (minimum, maximum). *P* values were calculated using Kruskal-Wallis test

*Abbreviations: BMI* body mass index, *WC* waist circumference, *systolic BP* systolic blood pressure, *diastolic BP* diastolic blood pressure, *HOMA-IR* homeostatic model assessment of insulin resistance, *HOMA-β* homeostatic model assessment of β-cell function, *T2DM* type 2 diabetes mellitus, *TG* triglyceride, *TC* total cholesterol, *HDL-c*, high density lipoprotein cholesterol, *LDL-c* low density lipoprotein cholesterol

^a^ *P* < 0.025 for normal group vs. pre-diabetic group

^b^ *P* < 0.025 for normal group vs. type 2 diabetic group

^c^ *P* < 0.025 for pre-diabetic group vs. type 2 diabetic group

**Fig. 1:**
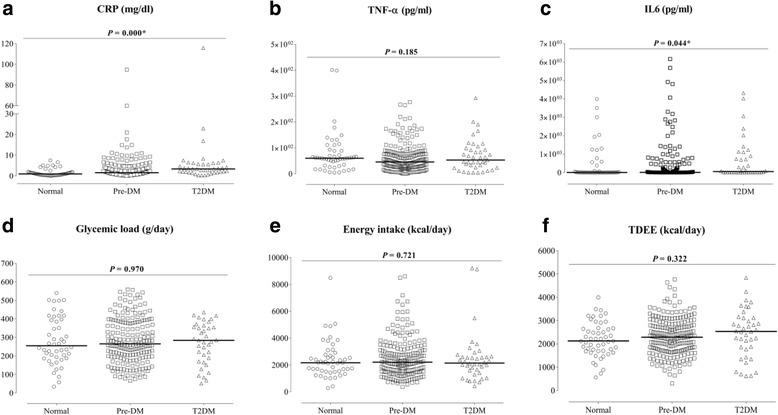
Levels of inflammatory markers, glycemic load, and type 2 diabetic risk factors of the normal, pre-diabetes, and type 2 diabetes mellitus (T2DM) groups. *P* values were calculated using Kruskal-Wallis test. Solid lines represent the median of each study group. **a**, **b**, and **c** show levels of C-reactive protein (CRP), tumor necrosis factor alpha (TNF-α), and interleukin 6 (IL6), respectively. **d**, **e**, and **f** show the daily consumed glycemic load (GL), daily energy intake, and total daily energy expenditure (TDEE), respectively

Levels of CRP & IL6 were all significantly higher in the T2DM group than in other groups (Fig. 1). However, no statistical differences were detected between groups in TNF-α levels, GL, or daily energy intake. Similarly, TDEE between study groups did not differ. Frequency of physical activity was similar among study groups, and correspondingly, muscle mass percentage was also similar.

The highest GL quartile (>381.03 g/d) showed a positive correlation with FBG, a T2DM clinical index (*r* = 0.289, *P* = 0.016). Noticeably, an acute-phase inflammatory protein, CRP, correlated with anthropometric indicators, including BMI (*r* = 0.332), WC (*r* = 0.363), BF (*r* = 0.373), VF (*r* = 0.320), and TrF (*r* = 0.362), *P* < 0.05 for all. It was also positively correlated with both systolic (*r* = 0.195) and diastolic blood pressure (*r* = 0.236). Considering lipid profiles, CRP correlated negatively with HDL-c (“good cholesterol”, *r* = −0.155), and positively with LDL-c (“bad cholesterol”, *r* = 0.124). CRP also showed correlation with the blood glucose indices 2hBG (*r* = 0.263) and HbA_1c_ (*r* = 0.259). Regarding insulin function, CRP significantly correlated with fasting insulin (*r* = 0.281), HOMA-IR (*r* = 0.299), and HOMA-β (*r* = 0.152). However, no relationship between CRP and the other inflammatory markers, TNF-α and IL6, was detected. TNF-α and IL6 were strongly correlated (*r* = 0.576, *P* = 0.000). Even though these two pro-inflammatory cytokines showed limited correlation with T2DM risk factors, both of them correlated with HOMA-β, the indicator of β-cell function (TNF-α: *r* = −0.200, *P* = 0.001 and IL6: *r* = −0.171, *P* = 0.003). Significant correlations were also found between TNF-α and insulin (*r* = −0.123, *P* = 0.035) and IL6 and FBG (*r* = 0.146, *P* = 0.012) (correlation data not shown).

The associations of inflammatory markers with T2DM were analyzed by logistic regression. CRP levels were divided according to suggested cut off values [26]. Since there are no standard cut off points for TNF-α and IL6, they were ranged by tertile. The results are presented in Table 3. A strongly increased risk of both pre-diabetes [OR (95% CI): 2.02 (1.09, 3.75)] and T2DM [OR (95% CI): 8.10 (2.74, 23.97)] was found in subjects with CRP levels ≥1 mg/dl. Likewise, subjects in the highest serum IL6 tertile had approximately a fourfold [OR (95% CI): 3.76 (1.33, 10.68)] higher risk of developing T2DM when compared to those in the lowest tertile. Subjects with TNF-α higher than 72.53 pg/ml also showed a trend towards increased risk of developing T2DM [OR (95% CI): 1.25 (0.42, 3.70)]. After adjusting for confounders, CRP and IL6 remained associated with increased risk of T2DM. Subjects with CRP levels ≥1 mg/dl and those in the highest IL6 level tertile had a markedly increased risk of T2DM [OR (95% CI): 7.51 (2.11, 26.74) and 4.95 (1.28, 19.11), respectively]. Although it did not reach statistical significance, a trend towards elevated risk of developing T2DM was observed in subjects with the highest TNF-α concentration range.

**Table 3:** Association between type 2 diabetic status and inflammatory markers

	Normal vs. pre-diabetic group				Normal vs. T2DM group			
	Crude OR (95% CI)	*P*	Adjusted OR^a^ (95% CI)	*P*	Crude OR (95% CI)	*P*	Adjusted OR^a^ (95% CI)	*P*
CRP (mg/dl)								
< 1.00	1		1		1		1	
≥ 1.00	2.02 (1.09,3.75)	0.026	1.89 (0.98,3.64)	0.059	8.10 (2.74, 23.97)	0.000	7.51 (2.11,26.74)	0.002
TNF-α (pg/ml)								
< 30.23	1		1		1		1	
30.23–72.53	0.40 (0.18, 0.85)	0.018	0.37 (0.17,0.81)	0.013	0.61 (0.21,1.74)	0.357	0.67 (0.18,2.46)	0.548
> 72.53	0.77 (0.33,1.77)	0.533	0.78 (0.33, 1.84)	0.563	1.25 (0.42,3.70)	0.691	1.56 (0.39,6.14)	0.528
IL6 (pg/ml)								
< 6.14	1		1		1		1	
6.14–61.91	1.54 (0.75,3.18)	0.242	1.66 (0.77,3.54)	0.192	1.68 (0.56,5.01)	0.351	3.22 (0.82,12.68)	0.094
> 61.91	1.64 (0.76,3.53)	0.206	1.88 (0.85,4.18)	0.122	3.76 (1.33,10.68)	0.013	4.95 (1.28,19.11)	0.020

*Abbreviations: CRP* C-reactive protein, *TNF-α* tumor necrosis factor alpha, *IL6* interleukin 6, *T2DM* type 2 diabetes mellitus

^a^OR was adjusted for age, gender, and body mass index

## Discussion

The present cross-sectional study focused on the relationship between T2DM, the most common type of diabetes, and its risk factors and markers among a group of working age people in a rural area.

There were no differences in socioeconomic characteristics and markers of unhealthy lifestyle (such as physical inactivity, smoking, and drinking alcohol) between the normal, pre-diabetic, and T2DM groups. Subjects self-reported that their work activities and housework reached the recommended daily physical activity level. The collected data suggest that most subjects were health conscious, as more than 70% did not smoke and half did not drink alcohol, with those who consumed alcohol only reporting low consumption. Lifestyle, health habits, and socioeconomic background were similar among three study groups. Baseline anthropometric and clinical characteristics were compared to determine any differences related to blood glucose levels. BMI, WC, BF, VF, TrF, blood pressure, HOMA-IR, and HOMA-β are recognized as risk factors for T2DM. We found that these factors were significantly higher in the pre-diabetic and T2DM groups than the normal group, with the highest levels observed in the T2DM group.

Previous studies have suggested a role for GL in T2DM development [27, 28]. Bhupathiraju et al. reported a higher risk of T2DM in those with the highest GL quintile [27]. Similarly, an increased risk of diabetes incidence among Caucasian men and all women except for Japanese Americans was significantly related to the highest GL quintile [28]. However, this association is still inconsistently observed [29, 30]. Results from the present study indicated that greater GL was associated with higher FBG levels. This is consistent with the hypothesis that GL is a risk factor for T2DM. Those who frequently consume a high GL diet may have a higher risk of developing the disease than those who do not. When investigating inflammatory markers, some studies have reported a positive association between GL and baseline CRP [30]. The relationships between GL and the inflammatory markers measured in this study, however, were ambiguous.

As a possible role of inflammatory markers in interrupting the insulin signaling pathway has been suggested, the present study analyzed the relationships of CRP, TNF-α, and IL6 with T2DM. The link between CRP and diabetes has been widely reported [5, 6]. We found that the pre-diabetic and T2DM groups had markedly higher CRP levels than the normal group. Subjects with high CRP levels had 2.2 and 8.1 times elevated risk of pre-diabetes and T2DM, respectively. Even after adjusting for confounders (i.e. age, gender, and BMI) this strong association remained. Therefore, the results of the present study support a role for CRP as an initiated marker for T2DM. Moreover, CRP has been used to forecast the risk of coronary heart disease [31], suggesting that there may be a positive link between abnormal blood sugar levels and coronary heart disease. However, no association between CRP vs TNF-α and CRP vs IL6 were detectable.

A high level of TNF-α is believed to induce insulin resistance and is considered to contribute to the development of diabetes. In the present study, although most T2DM risk factors were not related to TNF-α, strong relationships were observed between TNF-α and both insulin and HOMA-β. This is consistent with the results of an in vitro study which previously reported in 2008 that secretion of TNF-α can reduce the production of insulin released from the pancreatic β-cell [12]. In 2013, Costagliola also reported that, after adjusting for confounders, increased levels of TNF-α (13.5 pg/ml) were associated with severity of diabetic complications [32]. In the present study, high TNF-α level had a trend towards a 1.75 times increased risk of T2DM, although this did not reach statistical significance. Thus, TNF-α could possibly play a substantial role in the prolonged development of T2DM.

While the concentration of serum IL6 normally fluctuates according to physiological conditions and rapidly returns to basal levels, it becomes chronically elevated with T2DM [33]. In the present study, IL6 levels between study groups were significantly different. The T2DM group had the highest IL6 levels, and levels were also elevated above the normal group in the pre-diabetic group. Additionally, after adjusting for age, gender, BMI, BF, VF, TrF, and TG, subjects in the highest tertile of IL6 level had considerably increased risk (5.18 times) of developing T2DM when compared to those in the lowest tertile. The association between elevated IL6 levels and T2DM was also shown in a study by Wang (relative risk (95% CI): 1.31 (1.17, 1.46)) [16]. Popko et al. reported that IL6 concentrations were significantly higher in obese diabetic women than in women without diabetic symptoms [13], which is consistent with the findings of the present study.

## Conclusion

In summary, there was no difference in the appearance of all study subjects, and no complications were found. Our findings revealed that GL, the representative of high refined carbohydrate consumption, involved in T2DM. The inflammatory markers CRP, TNF-α, and IL6 were also associated with T2DM. These inflammatory markers may initiate T2DM. The study results showed a strong relationship between CRP and T2DM. Thus CRP may be a good marker for T2DM. T2DM is classified as a chronic disease that progresses over time and is influenced differently by many factors, including genetics and lifestyle. A limitation of this study is its cross-sectional design; while this enabled it to detect associations at a single time point, it could not provide information on complex longitudinal relationships. Therefore, a cohort study is planned in order to further clarify the associations detected in the present study. Moreover, individual differences such as gene polymorphisms could be planned for further study.

## Additional file

Additional file 1: Semi-food frequency questionnaire. The data contained in the questionnaire consists of the checklist of food and beverage, food portion size, and frequency of food intake (6 times/day, 4–5 times/day, 2–3 times/day, 1 time/day, 5–6 times/week, 2–4 times/week, 1 time/week, 1–3 times/month, and never). (DOC 410 kb)

## Abbreviations

2hBG: 2-h blood glucose
95% CI: 95% confidence intervals
BF: Body fat
BMI: Body mass index
CRP: C-reactive protein
ELISA: Enzyme linked immunosorbent assay
FBG: Fasting blood glucose
GL: Glycemic load
HbA1c: Glycated hemoglobin
HDL-c: High density lipoprotein cholesterol
HOMA-IR: The homeostatic model assessment of insulin resistance
HOMA-β: The homeostatic model assessment of beta cell function
IL6: Interleukin 6
LDL-c: Low density lipoprotein cholesterol
MET: Metabolic equivalent of task
OGTT: Oral glucose tolerance test
Semi-FFQ: Semi-quantitative food frequency questionnaire
T2DM: Type 2 diabetes mellitus
TC: Total cholesterol
TDEE: Total daily energy expenditure
TG: Triglyceride
TNF-α: Tumor necrosis factor alpha
TrF: Trunk fat
VF: Visceral fat
WC: Waist circumference
