# Supplementary material for: Elevated C-reactive protein, interleukin 6, tumor necrosis factor alpha and glycemic load associated with type 2 diabetes mellitus in rural Thais: a cross-sectional study
Source: BMC Endocr Disord. 2017 Jul 17;17:44. doi: 10.1186/s12902-017-0189-z (PMC5512726; doi:10.1186/s12902-017-0189-z)
Supplement: Additional file 1: — Semi-food frequency questionnaire. The data contained in the questionnaire consists of the checklist of food and beverage, food portion size, and frequency of food intake (6 times/day, 4–5 times/day, 2–3 times/day, 1 time/day, 5–6 times/week, 2–4 times/week, 1 time/week, 1–3 times/month, and never). (DOC 410 kb) [file 12902_2017_189_MOESM1_ESM.doc]

## **Semi-Food Frequency Questionnaire**

Date..............................

No. ............................... Age................ Tel...........................

| Food list | | Amount (compare with portion size) | | | Frequency | | | | | | | | | | | | | g/d | code |
| --- | --- | --- | --- | --- | --- | --- | --- | --- | --- | --- | --- | --- | --- | --- | --- | --- | --- | --- | --- |
|  |  |  |  |  | Times/day | | | | | | Times/week | | | | | Times/month | Never |  |  |
|  |  | Less | Equal | More | 6 | 4-5 | | 2-3 | | 1 | 5-6 | 2-4 | 1 | | | 1-3 | <1/mo |  |  |
| **Rice and starch *** Oil = ZT04802 | | | | | | | | | | | | | | | | | | | |
| Rice (1 plate = 195 g) | |  |  |  |  |  | |  | |  |  |  |  | | |  |  |  | ZT00014 |
| Rice soup (1 plate = 200 g) | |  |  |  |  |  | |  | |  |  |  |  | | |  |  |  | ZT00025 |
| Fried rice (1 plate = 195 g) + Oil 10% | |  |  |  |  |  | |  | |  |  |  |  | | |  |  |  |  |
| Glutinous rice (1 plate = 170 g) | |  |  |  |  |  | |  | |  |  |  |  | | |  |  |  | ZT00017 |
| Rice noodle (1 plate = 130 g) | |  |  |  |  |  | |  | |  |  |  |  | | |  |  |  |  |
| Fried rice noodle (1 plate = 130g) + Oil 10% | |  |  |  |  |  | |  | |  |  |  |  | | |  |  |  | ZT00002 |
| Instant noodle (Mama) (1 pack=110 g) | |  |  |  |  |  | |  | |  |  |  |  | | |  |  |  | ZT00027 |
| Rice noodle (round) (2 lump = 130 g) | |  |  |  |  |  | |  | |  |  |  |  | | |  |  |  | Z000018 |
| Bread (1 piece = 30 g) | |  |  |  |  |  | |  | |  |  |  |  | | |  |  |  | ZT00032 |
| Macaroni/Spaghetti (1 plate = 100 g) | |  |  |  |  |  | |  | |  |  |  |  | | |  |  |  | ZT00033 |
| Vegetable oil % (Rice 100 g=Oil 10 g) | |  |  |  |  |  | |  | |  |  |  |  | | |  |  |  | ZT04802 |
| **Meat, poultry, fish and sea food** | | | | | | | | | | | | | | | | | | | |
| Pork (no fat) | Boil/Grill |  |  |  |  |  | |  | |  |  |  |  | | |  |  |  | Z000062 |
| 6-7 pieces (30 g) | Fried |  |  |  |  |  | |  | |  |  |  |  | | |  |  |  | Z000063 |
| Pork (fat) | Boil/Grill |  |  |  |  |  | |  | |  |  |  |  | | |  |  |  | Z000064 |
| 6-7 pieces (30 g) | Fried |  |  |  |  |  | |  | |  |  |  |  | | |  |  |  | Z000065 |
| Cow meat (no fat) | Boil/Grill |  |  |  |  |  | |  | |  |  |  |  | | |  |  |  | ZT02417 |
| 6-7 pieces (30 g) | Fried |  |  |  |  |  | |  | |  |  |  |  | | |  |  |  | Z000059 |
| Cow meat (fat) | Boil/Grill |  |  |  |  |  | |  | |  |  |  |  | | |  |  |  | Z000060 |
| 6-7 pieces (30 g) | Fried |  |  |  |  |  | |  | |  |  |  |  | | |  |  |  | Z000061 |
| Chicken (thigh) | Boil/Grill (70g) |  |  |  |  |  | |  | |  |  |  |  | | |  |  |  | Z000068 |
| 1 piece | Fried (60 g) |  |  |  |  |  | |  | |  |  |  |  | | |  |  |  | Z000069 |
| Chicken (wing) | Boil/Grill (40 g) |  |  |  |  |  | |  | |  |  |  |  | | |  |  |  | Z000070 |
| 1 piece | Fried (30g) |  |  |  |  |  | |  | |  |  |  |  | | |  |  |  | Z000071 |
| Food list | | Amount (compare with portion size) | | | Frequency | | | | | | | | | | | | | g/d | code |
|  |  |  |  |  | Times/day | | | | | | Times/week | | | | | Times/month | Never |  |  |
|  |  | Less | Equal | More | 6 | 4-5 | | 2-3 | | 1 | 5-6 | 2-4 | 1 | | | 1-3 | <1/mo |  |  |
| Chicken (breast) | Boil/Grill (40 g) |  |  |  |  |  | |  | |  |  |  |  | | |  |  |  | Z000072 |
| 6-7 pieces | Fried (40g) |  |  |  |  |  | |  | |  |  |  |  | | |  |  |  | Z000073 |
| Chicken (skin) 6-7 pieces (30g) | |  |  |  |  |  | |  | |  |  |  |  | | |  |  |  | ZT02469 |
| Duck 6-7 pieces (40g) | |  |  |  |  |  | |  | |  |  |  |  | | |  |  |  | ZT02402 |
| Crab 30 g |  |  |  |  |  |  | |  | |  |  |  |  | | |  |  |  | ZT02429 |
| 6-7 Shrimp, 30 g |  |  |  |  |  |  | |  | |  |  |  |  | | |  |  |  | ZT02419 |
| 12-15 Shell fish, 30 g | |  |  |  |  |  | |  | |  |  |  |  | | |  |  |  | ZT02465 |
| Fresh water fish 1-2 pieces (40g) | |  |  |  |  |  | |  | |  |  |  |  | | |  |  |  | ZT02408 |
| Marine fish 1-2 pieces (40g) | |  |  |  |  |  | |  | |  |  |  |  | | |  |  |  | Z000048 |
| Canned fish 1-2 pieces (50g) | |  |  |  |  |  | |  | |  |  |  |  | | |  |  |  | ZT02449 |
| Squid 8-9 pieces, 30 g | |  |  |  |  |  | |  | |  |  |  |  | | |  |  |  | ZT02452 |
| Omelet (1 egg =50g) + Oil 10 % | |  |  |  |  |  | |  | |  |  |  |  | | |  |  |  | ZT02801 |
| Fried egg (1 egg= 50g) + Oil 5 % | |  |  |  |  |  | |  | |  |  |  |  | | |  |  |  |  |
| Boiled egg (1 egg = 50g) | |  |  |  |  |  | |  | |  |  |  |  | | |  |  |  |  |
| Egg in fried noodle/rice  (1 egg = 50g) | |  |  |  |  |  | |  | |  |  |  |  | | |  |  |  |  |
| Vegetable oil % | |  |  |  |  |  | |  | |  |  |  |  | | |  |  |  | ZT04802 |
| **Milk and dairy product** | | | | | | | | | | | | | | | | | | | |
| Milk (high calcium) (1 box = 200 g) | |  |  |  |  |  | |  | |  |  |  |  | | |  |  |  | Z000003 |
| Milk (UHT, sweetened) (1 box=200 g) | |  |  |  |  |  | |  | |  |  |  |  | | |  |  |  | Z000038 |
| Milk (UHT, whole fat) (1 box = 200 g) | |  |  |  |  |  | |  | |  |  |  |  | | |  |  |  | ZT02806 |
| Milk (Reduced fat) (1 box = 200 g) | |  |  |  |  |  | |  | |  |  |  |  | | |  |  |  | Z000022 |
| Fermented milk (1 box = 200 g) | |  |  |  |  |  | |  | |  |  |  |  | | |  |  |  | L453011 |
| Soy milk, whole fat (1 box = 200 g) | |  |  |  |  |  | |  | |  |  |  |  | | |  |  |  | ZT02805 |
| Milk, condensed, sweetened (1 teaspoon = 7 g) | |  |  |  |  |  | |  | |  |  |  |  | | |  |  |  | ZT02807 |
| Milo/Ovaltine (1 teaspoon = 3g) | |  |  |  |  |  | |  | |  |  |  |  | | |  |  |  | ZT04002 |
| Coffeemate (1 teaspoon = 3g) | |  |  |  |  |  | |  | |  |  |  |  | | |  |  |  | ZT04806 |
| Others | |  |  |  |  |  | |  | |  |  |  |  | | |  |  |  |  |
| Food list | | Amount (compare with portion size) | | | Frequency | | | | | | | | | | | | | g/d | code |
|  |  |  |  |  | Times/day | | | | | | Times/week | | | | | Times/month | Never |  |  |
|  |  | Less | Equal | More | 6 | 4-5 | | 2-3 | | 1 | 5-6 | 2-4 | 1 | | | 1-3 | <1/mo |  |  |
| **Vegetable** | | | | | | | | | | | | | | | | | | | |
| Morning glory  4 tablespoon | Fresh (25 g) |  |  |  |  |  | |  | |  |  |  |  | | |  |  |  | ZT01699 |
|  | Boil/Steam(50g) |  |  |  |  |  | |  | |  |  |  |  | | |  |  |  |  |
|  | Fried (50 g) |  |  |  |  |  | |  | |  |  |  |  | | |  |  |  |  |
| Ivy gourd (Boil, Steam) (4 tbsp=8g ) | |  |  |  |  |  | |  | |  |  |  |  | | |  |  |  | ZT01618 |
| White radish  4 tablespoon | Fresh (25 g) |  |  |  |  |  | |  | |  |  |  |  | | |  |  |  | ZT01631 |
|  | Boil/Steam(50g) |  |  |  |  |  | |  | |  |  |  |  | | |  |  |  |  |
|  | Fried (50 g) |  |  |  |  |  | |  | |  |  |  |  | | |  |  |  |  |
| Unripe papaya  1 plate | Fresh (70 g) |  |  |  |  |  | |  | |  |  |  |  | | |  |  |  |  |
|  | Soup/Fried (25g) |  |  |  |  |  | |  | |  |  |  |  | | |  |  |  |  |
| Bamboo shoot  4 tablespoon | Boil/Steam (25g) |  |  |  |  |  | |  | |  |  |  |  | | |  |  |  | ZT01628 |
|  | Fried (50 g) |  |  |  |  |  | |  | |  |  |  |  | | |  |  |  |  |
| Cucumber  4 pieces (50 g) | fresh (50 g) |  |  |  |  |  | |  | |  |  |  |  | | |  |  |  | ZT01620 |
|  | Boil/Steam(40g) |  |  |  |  |  | |  | |  |  |  |  | | |  |  |  |  |
|  | Fried (40 g) |  |  |  |  |  | |  | |  |  |  |  | | |  |  |  |  |
| Banana bud  4 tablespoon | Fresh (30 g) |  |  |  |  |  | |  | |  |  |  |  | | |  |  |  | ZT01661 |
|  | Boil/Steam/Soup (50 g) |  |  |  |  |  | |  | |  |  |  |  | | |  |  |  |  |
| 1 Thai eggplant = 10 g | Fresh |  |  |  |  |  | |  | |  |  |  |  | | |  |  |  | ZT01676 |
|  | Boil/Steam/Soup |  |  |  |  |  | |  | |  |  |  |  | | |  |  |  |  |
|  | Fried |  |  |  |  |  | |  | |  |  |  |  | | |  |  |  |  |
| Cabbage  4 tablespoon | Fresh (25g) |  |  |  |  |  | |  | |  |  |  |  | | |  |  |  | ZT01601 |
|  | Boil/Steam (50g) |  |  |  |  |  | |  | |  |  |  |  | | |  |  |  |  |
|  | Fried (50g) |  |  |  |  |  | |  | |  |  |  |  | | |  |  |  |  |
| Snake luffa  4 tablespoon | Fresh (25g) |  |  |  |  |  | |  | |  |  |  |  | | |  |  |  | ZT01629 |
|  | Boil/Steam (50g) |  |  |  |  |  | |  | |  |  |  |  | | |  |  |  |  |
|  | Fried (50g) |  |  |  |  |  | |  | |  |  |  |  | | |  |  |  |  |
| Sesban (Boil/Steam) (4 tbsp = 40g) | |  |  |  |  |  | |  | |  |  |  |  | | |  |  |  | ZT01613 |
| Food list | | Amount (compare with portion size) | | | Frequency | | | | | | | | | | | | | g/d | code |
|  |  |  |  |  | Times/day | | | | | | Times/week | | | | | Times/month | Never |  |  |
|  |  | Less | Equal | More | 6 | 4-5 | | 2-3 | | 1 | 5-6 | 2-4 | 1 | | | 1-3 | <1/mo |  |  |
| Pumpkin  4 tablespoon | Boil/Steam/Soup (35 g |  |  |  |  |  | |  | |  |  |  |  | | |  |  |  | ZT01638 |
|  | Fried (45 g) |  |  |  |  |  | |  | |  |  |  |  | | |  |  |  |  |
| White popinac (4 tablespoon = 10 g) | |  |  |  |  |  | |  | |  |  |  |  | | |  |  |  |  |
| Chinese kale  4 tablespoon | Fresh (15 g) |  |  |  |  |  | |  | |  |  |  |  | | |  |  |  | ZT01708 |
|  | Boil/Steam/Soup (30 g |  |  |  |  |  | |  | |  |  |  |  | | |  |  |  |  |
|  | Fried (35 g) |  |  |  |  |  | |  | |  |  |  |  | | |  |  |  |  |
| Yard long bean  4 tablespoon | Fresh (30 g) |  |  |  |  |  | |  | |  |  |  |  | | |  |  |  | ZT01625 |
|  | Boil/Steam/Soup (40g) |  |  |  |  |  | |  | |  |  |  |  | | |  |  |  |  |
|  | Fried (35 g) |  |  |  |  |  | |  | |  |  |  |  | | |  |  |  |  |
| Lettuce 4 tablespoon = 15g | |  |  |  |  |  | |  | |  |  |  |  | | |  |  |  | ZT01632 |
| Bitter bean  4 tablespoon | Fresh (40 g) |  |  |  |  |  | |  | |  |  |  |  | | |  |  |  | Z000033 |
|  | Fried (35 g) |  |  |  |  |  | |  | |  |  |  |  | | |  |  |  |  |
| Djenkol bean 4 tablespoon (45 g) | |  |  |  |  |  | |  | |  |  |  |  | | |  |  |  | Z000045 |
| Cashew nut  4 tablespoon | Fresh (40 g) |  |  |  |  |  | |  | |  |  |  |  | | |  |  |  | ZT01622 |
|  | Fried (35 g) |  |  |  |  |  | |  | |  |  |  |  | | |  |  |  |  |
| Others | |  |  |  |  |  | |  | |  |  |  |  | | |  |  |  |  |
|  | |  |  |  |  |  | |  | |  |  |  |  | | |  |  |  |  |
|  | |  |  |  |  |  | |  | |  |  |  |  | | |  |  |  |  |
|  | |  |  |  |  |  | |  | |  |  |  |  | | |  |  |  |  |
|  | |  |  |  |  |  | |  | |  |  |  |  | | |  |  |  |  |
|  | |  |  |  |  |  | |  | |  |  |  |  | | |  |  |  |  |
|  | |  |  |  |  |  | |  | |  |  |  |  | | |  |  |  |  |
|  | |  |  |  |  |  | |  | |  |  |  |  | | |  |  |  |  |
|  | |  |  |  |  |  | |  | |  |  |  |  | | |  |  |  |  |
|  | |  |  |  |  |  | |  | |  |  |  |  | | |  |  |  |  |
|  | |  |  |  |  |  | |  | |  |  |  |  | | |  |  |  |  |
| Food list | | Amount (compare with portion size) | | | Frequency | | | | | | | | | | | | | g/d | code |
|  |  |  |  |  | Times/day | | | | | | Times/week | | | | Times/month | | Never |  |  |
|  |  | Less | Equal | More | 6 | 4-5 | | 2-3 | | 1 | 5-6 | 2-4 | 1 | | 1-3 | | <1/mo |  |  |
| **Fruit** | |  |  |  |  |  | |  | |  |  |  |  | |  | |  |  |  |
| Tangerine (1 medium size = 140 g) | |  |  |  |  |  | |  | |  |  |  |  | |  | |  |  | ZT02014 |
| Banana (1 banana = 50 g) | |  |  |  |  |  | |  | |  |  |  |  | |  | |  |  | ZT02002 |
| Guava (9 small pieces = 100 g) | |  |  |  |  |  | |  | |  |  |  |  | |  | |  |  | ZT02040 |
| Ripe papaya (8 pieces = 100 g) | |  |  |  |  |  | |  | |  |  |  |  | |  | |  |  | ZT01644 |
| Pineapple (3 pieces = 100 g) | |  |  |  |  |  | |  | |  |  |  |  | |  | |  |  | ZT02023 |
| Apple (1 apple = 150 g) | |  |  |  |  |  | |  | |  |  |  |  | |  | |  |  | ZT02025 |
| Unripe mango (1/2 mango = 100 g) | |  |  |  |  |  | |  | |  |  |  |  | |  | |  |  | ZT02036 |
| Ripe mango (2-3 pieces = 60 g) | |  |  |  |  |  | |  | |  |  |  |  | |  | |  |  | ZT02029 |
| Durian (2 pieces = 100 g) | |  |  |  |  |  | |  | |  |  |  |  | |  | |  |  | Z000020 |
| Jackfruit (5 pieces = 100 g) | |  |  |  |  |  | |  | |  |  |  |  | |  | |  |  | ZT02049 |
| Mangosteen (2-3 mangosteens = 40 g) | |  |  |  |  |  | |  | |  |  |  |  | |  | |  |  |  |
| Lychee (2-3 Lychee = 30 g) | |  |  |  |  |  | |  | |  |  |  |  | |  | |  |  | ZT02046 |
| Rambutan (4-5 rambutan = 100 g) | |  |  |  |  |  | |  | |  |  |  |  | |  | |  |  | ZT02039 |
| Long-Kong/Langsad (8-10 pieces = 100 g) | |  |  |  |  |  | |  | |  |  |  |  | |  | |  |  | ZT02013 |
| Others | | | | | | | | | | | | | | | | | | | |
|  | |  |  |  |  |  | |  | |  |  |  |  |  | | |  |  |  |
|  | |  |  |  |  |  | |  | |  |  |  |  |  | | |  |  |  |
|  | |  |  |  |  |  | |  | |  |  |  |  |  | | |  |  |  |
|  | |  |  |  |  |  | |  | |  |  |  |  |  | | |  |  |  |
|  | |  |  |  |  |  | |  | |  |  |  |  |  | | |  |  |  |
|  | |  |  |  |  |  | |  | |  |  |  |  |  | | |  |  |  |
|  | |  |  |  |  |  | |  | |  |  |  |  |  | | |  |  |  |
|  | |  |  |  |  |  | |  | |  |  |  |  |  | | |  |  |  |
|  | |  |  |  |  |  | |  | |  |  |  |  |  | | |  |  |  |
|  | |  |  |  |  |  | |  | |  |  |  |  |  | | |  |  |  |
|  | |  |  |  |  |  | |  | |  |  |  |  |  | | |  |  |  |
|  | |  |  |  |  |  | |  | |  |  |  |  |  | | |  |  |  |
| Food list | | Amount (compare with portion size) | | | Frequency | | | | | | | | | | | | | g/d | code |
|  |  |  |  |  | Times/day | | | | | | Times/week | | | Times/month | | | Never |  |  |
|  |  | Less | Equal | More | 6 | 4-5 | | 2-3 | | 1 | 5-6 | 2-4 | 1 | 1-3 | | | <1/mo |  |  |
| **Sugar** | | | | | | | | | | | | | | | | | | | |
| Sugar (1 teaspoon = 4 g) | |  |  |  |  |  | |  | |  |  |  |  |  | | |  |  | ZT04401 |
| - Add in diet or beverage | |  |  |  |  |  | |  | |  |  |  |  |  | | |  |  |  |
| - Jam (1 teaspoon = 7 g) | |  |  |  |  |  | |  | |  |  |  |  |  | | |  |  | ZT04006 |
| **Dessert** | | | | | | | | | | | | | | | | | | | |
| - Dessert with coconut milk (1 cup = 250 g) | |  |  |  |  |  | |  | |  |  |  |  |  | | |  |  | Z000031 |
| - Dessert without coconut milk (1 cup = 250g | |  |  |  |  |  | |  | |  |  |  |  |  | | |  |  | Z000023 |
| Deep fried banana (2-3 pieces = 60 g) | |  |  |  |  |  | |  | |  |  |  |  |  | | |  |  | Z000058 |
| Cake/Doughnut (1 piece = 80 g) | |  |  |  |  |  | |  | |  |  |  |  |  | | |  |  | Z00024 |
| Cookie/Biscuit/Oreo (1 piece = 10 g) | |  |  |  |  |  | |  | |  |  |  |  |  | | |  |  | ZT00022 |
| Fried potato (Lays) (1 pack = 20 g) | |  |  |  |  |  | |  | |  |  |  |  |  | | |  |  | Z000021 |
| Snack (1 pack = 30 g) | |  |  |  |  |  | |  | |  |  |  |  |  | | |  |  | Z000002 |
| Thai dessert (100 g) | |  |  |  |  |  | |  | |  |  |  |  |  | | |  |  |  |
| Caramelized crisp (100 g) | |  |  |  |  |  | |  | |  |  |  |  |  | | |  |  |  |
| Ice-cream (2 scoops = 50 g) | |  |  |  |  |  | |  | |  |  |  |  |  | | |  |  | ZT02809 |
| Others | |  |  |  |  |  | |  | |  |  |  |  |  | | |  |  |  |
|  | |  |  |  |  |  | |  | |  |  |  |  |  | | |  |  |  |
|  | |  |  |  |  |  | |  | |  |  |  |  |  | | |  |  |  |
|  | |  |  |  |  |  | |  | |  |  |  |  |  | | |  |  |  |
|  | |  |  |  |  |  | |  | |  |  |  |  |  | | |  |  |  |
|  | |  |  |  |  |  | |  | |  |  |  |  |  | | |  |  |  |
|  | |  |  |  |  |  | |  | |  |  |  |  |  | | |  |  |  |
|  | |  |  |  |  |  | |  | |  |  |  |  |  | | |  |  |  |
|  | |  |  |  |  |  | |  | |  |  |  |  |  | | |  |  |  |
|  | |  |  |  |  |  | |  | |  |  |  |  |  | | |  |  |  |
|  | |  |  |  |  |  | |  | |  |  |  |  |  | | |  |  |  |
|  | |  |  |  |  |  | |  | |  |  |  |  |  | | |  |  |  |
| Food list | | Amount (compare with portion size) | | | Frequency | | | | | | | | | | | | | g/d | code |
|  |  |  |  |  | Times/day | | | | | | Times/week | | | Times/month | | | Never |  |  |
|  |  | Less | Equal | More | 6 | 4-5 | | 2-3 | | 1 | 5-6 | 2-4 | 1 | 1-3 | | | <1/mo |  |  |
| Coconut milk (1 ladle = 60 g) | |  |  |  |  |  | |  | |  |  |  |  | |  | |  |  | ZT01702 |
| Coconut meat (1 tabelspoon = 10 g) | |  |  |  |  |  | |  | |  |  |  |  | |  | |  |  | ZT01234 |
| **Beverage** | | | | | | | | | | | | | | | | | | | |
| Tea/Coffee (1 glass = 250 ml) | |  |  |  |  |  | |  | |  |  |  |  | |  | |  |  | MU01050 |
| Fruit juice (1 glass = 200 g) | |  |  |  |  |  | |  | |  |  |  |  | |  | |  |  |  |
| Soft drink (1 bottle = 300 g) | |  |  |  |  |  | |  | |  |  |  |  | |  | |  |  | ZT04009 |
| Sweetened drink (1 glass = 200 g) | |  |  |  |  |  | |  | |  |  |  |  | |  | |  |  | ZT000015 |
| Spirits (1 glass = 60 cc) | |  |  |  |  |  | |  | |  |  |  |  | |  | |  |  |  |
| Beer (1 glass = 200 cc) | |  |  |  |  |  | |  | |  |  |  |  | |  | |  |  | ZT04013 |
| Others | |  |  |  |  |  | |  | |  |  |  |  | |  | |  |  |  |
|  | |  |  |  |  |  | |  | |  |  |  |  | |  | |  |  |  |
|  | |  |  |  |  |  | |  | |  |  |  |  | |  | |  |  |  |
|  | |  |  |  |  |  | |  | |  |  |  |  | |  | |  |  |  |
|  | |  |  |  |  |  | |  | |  |  |  |  | |  | |  |  |  |
|  | |  |  |  |  |  | |  | |  |  |  |  | |  | |  |  |  |
|  | |  |  |  |  |  | |  | |  |  |  |  | |  | |  |  |  |
|  | |  |  |  |  |  | |  | |  |  |  |  | |  | |  |  |  |
|  | |  |  |  |  |  | |  | |  |  |  |  | |  | |  |  |  |
| **Seasoning** | | | | | | | | | | | | | | | | | | | |
| Pickled fish (1 tablespoon = 15 g) | |  |  |  |  |  | |  | |  |  |  |  | |  | |  |  | ZT02457 |
| Fish sauce (1 teaspoon = 5 g) | |  |  |  |  |  | |  | |  |  |  |  | |  | |  |  | ZT03216 |
| **Beans** | | | | | | | | | | | | | | | | | | | |
| Cashew nut (1 tablespoon = 10 g) | |  |  |  |  |  |  | |  | |  |  |  | |  | |  |  | ZT01211 |
| Peanut (1/2 cup = 62.5 g) | |  |  |  |  |  |  | |  | |  |  |  | |  | |  |  | ZT01227 |
| Sugar pea (1 pack = 25 g) | |  |  |  |  |  |  | |  | |  |  |  | |  | |  |  |  |
